# The Prospective Analysis of Biomarkers in Sepsis: Correlation With Clinical Outcomes

**DOI:** 10.7759/cureus.70965

**Published:** 2024-10-06

**Authors:** Vaishnavi Reddy, Mahendra Wante, Dakshayani S Nirhale, Pragna Puvvada, Romi H Gaudani

**Affiliations:** 1 Department of General Surgery, Dr. D. Y. Patil Medical College, Hospital and Research Centre, Dr. D. Y. Patil Vidyapeeth (Deemed to be University), Pune, IND

**Keywords:** infection, morbidity and mortality, septic shock, serum biomarkers, severe sepsis

## Abstract

Background

Sepsis is a medical emergency and necessitates immediate diagnosis and treatment to prevent the progression to severe sepsis, septic shock, and potentially mortality.

Aim

This study aims to study the diagnostic significance of conventional and new markers, interleukin-10 (IL-10), in predicting the severity of sepsis.

Methodology

A prospective observational study was conducted in the department of surgery in a tertiary care hospital in Pune, India. The study included 100 patients diagnosed with a quick Sequential Organ Failure Assessment (qSOFA) score of ≥2. Serum C-reactive protein (CRP), procalcitonin (PCT), and interleukin-10 (IL-10) levels were measured. Receiver operating characteristic (ROC) curves were plotted to assess the diagnostic performance of these biomarkers.

Results

The mean serum CRP level on day 7 was significantly higher than the baseline, day 1, and day 3 groups (p=0.0001). On analysis by repeated measure, the ANOVA test revealed that the mean CRP levels on day 7 were significantly higher. The mean PCT levels on day 7, day 3, and day 1 groups were significantly lower than those on day 1, day 2, and day 2, respectively (p=0.0001). The mean p-value of 3.3 g/L CRP was significantly lower on day 1 than that on day 3. IL-10 levels showed a significant upward trend, rising from 5.21 pg/mL at baseline to 7.57 pg/mL by day 7, with a p-value of <0.0001. Our cohort population showed elevated IL-10 values on the day of admission in a total of 15 patients. In our study, we observed that 11 patients with elevated IL-10 levels progressed toward multiple organ dysfunction syndrome (MODS) and four mortalities. IL-10 is a crucial marker for identifying patients with worsening surgical sepsis.

Conclusion

IL-10, CRP, and PCT have potential as prognostic markers in assessing and predicting disease severity. The dynamic changes in these biomarkers correlate strongly with clinical outcomes, suggesting their role in guiding treatment decisions.

## Introduction

Sepsis, an extensive disorder resulting from an abnormal immune response to infection, continues to be a significant contributing factor to morbidity and mortality worldwide [1]. Over time, the concept of sepsis has undergone significant changes. In 1904, William Osler made a crucial observation, noting that fatalities were often due to the body's response to infections rather than the infections themselves. He emphasized that the body's response to infection sustains sepsis by affecting complement, coagulation cascades, and endothelial function [2]. According to data released in 2020 by the World Health Organization (WHO), there were 11 million sepsis-related fatalities and 48.9 million cases globally, accounting for 20% of all deaths [1]. In India, sepsis affects approximately 11.3 million people with a mortality rate of 2.9 million [3]. Bacteria are the primary causative agents, although viruses, parasites, and fungi can also be responsible. These pathogens enter the bloodstream, propagate, release virulence factors, activate endogenous mediators, and trigger an immune response that can damage host tissues and organs [4].

Severe sepsis can lead to organ dysfunction and hypoperfusion, potentially progressing to septic shock, characterized by hypotension [5]. The clinical presentation of sepsis varies, complicating diagnosis and treatment. Early detection, diagnosis, and treatment are critical for improving outcomes, necessitating a multidisciplinary approach [5,6]. Delayed recognition and treatment can result in adverse effects on multiple organs. The early initiation of antimicrobial therapy is essential for optimal outcomes [5]. Research efforts have focused on identifying biomarkers for early sepsis detection, such as cytokines, marker cells, receptors, and indicators of vascular endothelial damage and organ failure. Advances in molecular biology have provided potential early biomarkers during the acute stage of sepsis. Despite this, the diagnosis of sepsis remains complex, involving comprehensive clinical assessments and laboratory testing [7]. In surgical patients, sepsis accounts for nearly one-third of all cases, with intra-abdominal infections being a common cause [8]. Prompt diagnosis, aggressive treatment, infection control, and early recognition are crucial. Preventing sepsis during the postoperative period is paramount.

Procalcitonin (PCT) and C-reactive protein (CRP) are potential biomarkers frequently studied. PCT has shown greater utility than CRP in diagnosing, prognosing, and differentiating viral from bacterial infections [9]. Another such marker is interleukin-10 (IL-10). Interleukin-10 (IL-10) is a crucial anti-inflammatory cytokine that regulates immune responses, preventing excessive tissue damage and maintaining immune balance. Produced by various immune cells, IL-10 suppresses pro-inflammatory cytokines such as tumor necrosis factor-alpha (TNF-α), IL-1, and IL-6, limiting inflammation. In sepsis, IL-10 plays a dual role: it reduces harmful inflammation but can also impair immune responses, contributing to poor outcomes. Neutralizing IL-10 in animal models of sepsis increases inflammatory cytokine production and mortality, while administering recombinant IL-10 offers therapeutic benefits. IL-10 is pivotal in modulating immune responses, making it essential in the context of sepsis management.

We aimed to study the diagnostic significance of conventional (PCT and CRP) and new (IL-10) markers in accurately identifying sepsis and assessing its progression toward irreversible sepsis, shock, and multiorgan dysfunction.

## Materials and methods

The department of surgery at a tertiary care hospital in Pune conducted a prospective observational study. The study included 100 patients diagnosed with sepsis based on the Sepsis-3 criteria [10]. The study included patients of either sex above 21 years and admitted with a clinical diagnosis of sepsis and a quick Sequential Organ Failure Assessment (qSOFA) score of 2 or more.

Patients under the age of 18 were not included in the study. Furthermore, subjects with aplasia or preexisting immunosuppression were excluded. Furthermore, subjects who were in remission from a hematological disease or solid tumor, were undergoing chemotherapy within five years prior to inclusion, had an inherent immune deficit, or were undergoing extracorporeal circulation within one month prior to inclusion were also excluded.

We screened patients who were attending the department of surgery and admitted to the intensive care unit (ICU), providing detailed information about the study to those eligible for participation. The patients were screened consecutively as they were admitted during the study period of two years.

Those willing to participate signed an informed written consent. The Institutional Ethics Sub-Committee of Dr. D. Y. Patil Medical College, Hospital and Research Centre approved the study before enrolment (reference number: I.E.S.C/327/2022).

We subjected the study population to a thorough clinical examination and laboratory evaluation. We collected serial blood samples on the day of admission, 24 hours later (day 1), day 3, and day 7 to assess the pattern of change in biomarker values as the disease progressed. We used immunoturbidimetric assays to measure CRP levels, immunoluminometric assays to measure PCT levels, and enzyme-linked immunosorbent assays (ELISA) to measure IL-10 levels. The patients received broad-spectrum antibiotics as per the standard operating procedure (SOP) antibiotic protocol as per institutional policy of antibiotics and supportive management. The standard operating protocol of antibiotics in the institution states the following: the use of third-generation cephalosporins for clean surgical cases, the combination of cephalosporins and beta-lactamase inhibitors, or the addition of aminoglycosides in clean contaminated cases. In accordance with the disease progression, total leukocyte count, culture sensitivity reports, and sepsis biomarkers, higher antibiotics may be administered.

Statistical analysis

We used the SPSS software (IBM SPSS Statistics, Armonk, NY) to conduct statistical analyses. We used Student's t-test to compare continuous variables, expressed as mean±SD. We used the chi-square test to compare the categorical variables. In order to assess the diagnostic efficacy of CRP, PCT, and IL-10, we generated receiver operating characteristic (ROC) curves.

## Results

Among the 100 patients, the majority of patients (54, 54%) were in the age group of 41-50 years, with more male patients (56, 56%) being affected. The mean age was 41.08±10.26 years, ranging from 21 to 59 years. However, age did not affect the incidence of sepsis. Sepsis spread was highest in the patients with postoperative infections (28%), including both surgical site infections (SSIs) and deep tissue space infections, followed by polytrauma (20%) and peritonitis (16%), while pelvic inflammatory disease (3%) was the least common diagnosis.

In the study population, the total leukocyte count (TLC) ranged from 12.3 to 30.4×10³/mm³, with a mean of 17.75±5.72. The majority of patients (80%) had a qSOFA score of >2, ranging from 0 to 22, and a mean of 11.07±6.95.

We measured the CRP levels at baseline and over subsequent days (day 1, day 3, and day 7) in patients with sepsis. Despite minor fluctuations, the mean CRP levels remained relatively stable throughout the observation period (25.82 mg/L at baseline to 24.86 mg/L at day 7), with no statistically significant differences noted (p=0.974). This suggests that CRP may not be an ideal marker for acute changes in sepsis severity. Repeated analysis by ANOVA revealed no significant decrease in CRP (p=0.974) (Table 1).

**Table 1:** Variation in C-reactive protein (CRP) Data expressed as mean (±SD) and p-value by chi-square test

CRP (mg/L)	Mean±SD	P-value
Baseline	25.82±26.26	0.974
Day 1	25.55±17.15	
Day 3	25.01±22.97	
Day 7	24.86±24.70	

**Figure 1:**
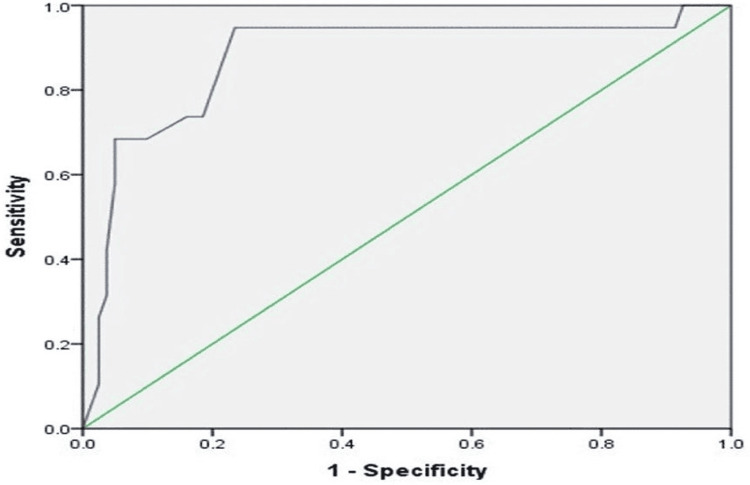
Receiver operating characteristic (ROC) curve analysis of CRP level in patients with sepsis CRP: C-reactive protein

At a cutoff value of 9.35 mg/L, serum CRP level had a sensitivity of 89.5% and a specificity of 99.7% (area under the curve {AUC}, 0.878; 95% CI, 0.779-0.977) (Figure 1).

On analysis by repeated measure, the ANOVA test revealed that the mean procalcitonin on day 7 was significantly higher than the baseline, day 1, and day 3 groups (p<0.0001). Moreover, post hoc analysis by Bonferroni's multiple comparison tests revealed a significant increase in mean procalcitonin from baseline to days 3 and 7 (p-value of <0.0001 both) and between days 3 and 7 (p-value of <0.0001) (Table 2).

**Table 2:** Variation in procalcitonin level Data expressed as mean and p-value by chi-square test

Procalcitonin (µg/L)		
	Mean±SD	P-value
Baseline	26.25±17.36	<0.0001
Day 1	26.39±16.61	
Day 3	35.45±19.94	
Day 7	44.27±18.20	

**Figure 2:**
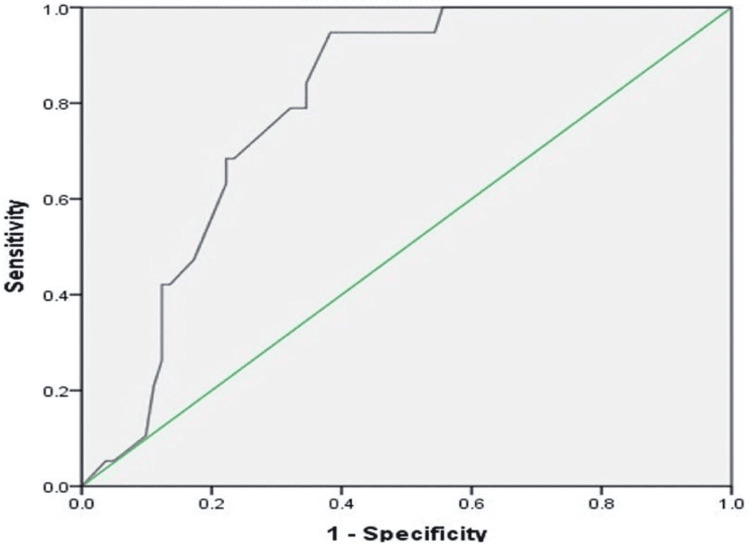
Receiver operating characteristic (ROC) curve analysis of procalcitonin level in patients with sepsis

At a cutoff value of 16.75 µg/L, serum procalcitonin level had a sensitivity of 78.9% and a specificity of 68.9% (AUC, 0.793; 95% CI, 0.704-0.882) (Figure 2).

On analysis by repeated measure, the ANOVA test revealed that the mean interleukin-10 of the day 7 group was significantly increased than the baseline, day 1, and day 3 groups (p<0.0001). Moreover, post hoc analysis by Bonferroni's multiple comparison tests revealed a significant increase in mean interleukin-10 from baseline to days 3 and 7 (p-value of 0.019 and <0.0001, respectively) and between days 3 and 7 (p-value of 0.014) (Table 3).

**Table 3:** Variation in IL-10 levels Data expressed as mean and p-value by chi-square test

Interleukin-10 (IL-10) (pg/mL)		
	Mean±SD	P-value
Baseline	5.21±3.28	<0.0001
Day 1	5.34±2.63	
Day 3	6.53±3.16	
Day 7	7.57±3.92	

**Figure 3:**
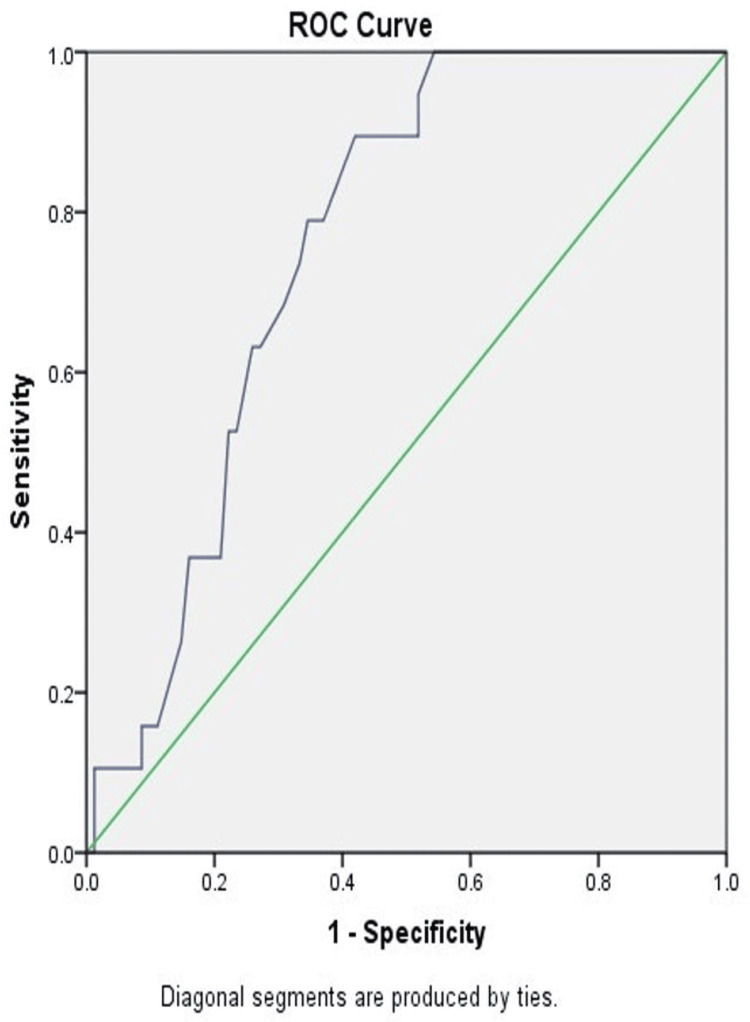
Receiver operating characteristic (ROC) curve analysis of interleukin-10 level in patients with sepsis

At a cutoff value of 3.3 µg/L, the interleukin-10 level had a sensitivity of 78.9% and a specificity of 65.4% (AUC, 0.757; 95% CI, 0.661-0.853; p-value, 0.001) (Figure 3).

Antibiotics were administered for ≤7 days in the majority of patients (55%), followed by 41% of patients for 8-14 days (41%). The mean duration ranged from four to 15 days with a mean of 7.89±2.80 days. The fluctuating biomarker values were used as an indicative marker in gauging for appropriate response to antibiotics.

The majority of the patients improved (64%), while 28% progressed to multiorgan dysfunction, and there were eight fatalities in our study cohort (Table 4).

**Table 4:** Prognostic outcomes of patients with sepsis (N=100) Data expressed in frequency (n) and percentage (%) MODS: multiple organ dysfunction syndrome

Outcome	N (%)
Death	8 (8)
Progress to MODS	28 (28)
Improved	64 (64)

## Discussion

Sepsis is an organ dysfunction that has the potential to be fatal and is caused by an infection-induced unregulated host response. It is a complex and severe syndrome that arises as a result of the body's response to an infection, which results in tissue and organ injury [3]. We recognize sepsis as a medical emergency that necessitates immediate diagnosis and treatment to prevent the progression to severe sepsis, septic shock, and potentially fatality. The diagnosis of sepsis is challenging because there are no definitive results from laboratory or radiographic examinations [4]. According to a recent meta-analysis, sepsis is the primary cause of hospital admissions and ICU stays, which places a significant burden on healthcare systems worldwide. Direct costs, such as prolonged hospital stays and treatments, and indirect costs, such as long-term disability and lost productivity, comprise the substantial economic burden of sepsis. In high-income countries, the financial impact is also considerable, despite the presence of a more sophisticated healthcare infrastructure and enhanced remedies [5].

Epidemiological studies currently show that sepsis affects a higher percentage of patients than in previous years. Recent data from the United States has demonstrated that sepsis is one of the most expensive conditions that necessitate hospital admissions. Reports range from 20% to 50% for fatality rates [6]. However, mortality statistics are inconsistent worldwide [6]. Surgical sepsis is a significant contributor to global healthcare costs, mortality, and morbidity. SSIs are among the most prevalent healthcare-associated infections, affecting up to 11% of patients in developed countries and as many as 20% in developing nations, according to the World Health Organization (WHO) [5]. The burden is disproportionately severe in low- and middle-income countries (LMICs) due to a scarce supply of resources, a higher prevalence of underlying health conditions, and deficient infection control practices. A multifaceted approach is required to alleviate the burden of surgical sepsis, which encompasses improved surgical techniques, stringent infection control practices, effective antimicrobial stewardship, and enhanced postoperative care.

Sociodemographic distribution of sepsis

The age group of 41-50 years was the most prevalent among patients with sepsis in our study, accounting for 46% of the participants. This discovery is consistent with other research that suggests a higher prevalence of sepsis in middle-aged and elderly adults. Sepsis incidence increased significantly with age, particularly among individuals over the age of 40, according to a study conducted in the United States [6]. Nasa et al., on the other hand, discovered that the average age of sepsis patients was 64 years, underscoring the regional demographic disparities [7]. Consistent with our study, an Indian study reported a mean age of 46.54±18.8 years among sepsis patients [8]. Sepsis affected males more frequently than females, accounting for 56% of our participants. An extensive review by Lakbar et al. discusses several studies reporting a higher prevalence of sepsis in males [9]. Differences in comorbid conditions, healthcare access, and biological responses to infections may influence the gender disparity [10]. Another study from India reported similar findings, indicating that 56.8% of sepsis patients were male [11].

Etiological factors

The most prevalent etiological factors identified in our study were postoperative surgical site infections (28%), as well as wound/diabetic foot infections (20%). Several studies have indicated that postoperative surgical sites are at a high risk of developing sepsis, highlighting the vulnerability of these areas to infection following surgery [12,13]. However, other studies have identified urosepsis as the most prevalent etiology for sepsis (25%), with others indicating the effect of the diversity of patient populations and healthcare settings [14,15].

Duration of antibiotics administered

Guidelines recommend a 7-10-day course of antibiotics for sepsis treatment [16]. The mean duration of antibiotic administration in our study was 7.89 days, which concurred with the guidelines. The use of culture sensitivity reports to customize antibiotic therapy is critical for preventing resistance and reducing superfluous, protracted antibiotic use.

Elevated total leukocyte count and qSOFA score in association with surgical sepsis

The body's inflammatory response to infection is a common finding in sepsis. In our study, we found a substantially elevated mean total leukocyte count (TLC) of 17.75±5.72. This finding is consistent with other research, including the Surviving Sepsis Campaign, which emphasizes leukocytosis as a prevalent indicator of sepsis [16,17].

Outside of intensive care settings, the qSOFA score serves as a simplified instrument for evaluating the severity of sepsis. The qSOFA score of 80% of the patients in our study was greater than 2, suggesting a higher probability of severe sepsis or septic shock. A meta-analysis and an additional study by the authors have validated the utility of qSOFA as a predictor of sepsis severity and mortality [3,18].

Biomarkers and their dynamics in surgical sepsis

Recent studies from 2022 and 2023 have demonstrated the use of procalcitonin and IL-10 in early sepsis diagnosis and disease severity assessment in surgical patients [19,20]. The dynamic changes of these biomarkers over time are consistent with our findings, thereby confirming their importance in patient management and clinical decision-making.

CRP levels demonstrated minimal fluctuations but remained relatively consistent, indicating that CRP may not accurately represent acute changes in sepsis severity or treatment response. Studies conducted worldwide, particularly in surgical contexts, reported similar limitations in the predictive utility of CRP in sepsis [21,22].

During the study, the substantial increase in procalcitonin levels correlated with both the severity of the disease and the efficacy of the treatment. This was consistent with research that demonstrated PCT's efficacy in steering antibiotic therapy and predicting outcomes in surgical sepsis patients [23,24].

IL-10 is an anti-inflammatory cytokine that is needed to predict the worst outcomes of sepsis, such as multiple organ dysfunction syndrome (MODS) and immune system modulation. Similar to other studies, the elevated levels of IL-10 during the course of the study indicate its involvement in the sepsis response [25,26].

Clinical outcomes and prognostic indicators

The mean hospital stay in our study was 15.79 days, which is indicative of the variable clinical course of surgical sepsis and the impact of biomarkers on disease severity and recovery. Previous research has underscored the significance of early biomarker assessment in outcome prediction by demonstrating a correlation between extended hospital stays, higher initial qSOFA scores, and elevated biomarker levels [27,28]. Our research indicated that 64% of the patients experienced improvement, 28% advanced to MODS, and 8% passed away. This underscores the necessity of early intervention and monitoring, as well as the heterogeneous nature of sepsis outcomes in surgical settings.

Role of IL-10 in the diagnosis of MODS

IL-10 levels showed a significant upward trend, rising from 5.21 pg/mL at baseline to 7.57 pg/mL by day 7, with a p-value of <0.0001. Our cohort population showed elevated IL-10 values on the day of admission in a total of 15 patients. In our study, we observed 11 patients with elevated IL-10 levels progress toward MODS and four mortalities. IL-10 is a crucial marker for identifying patients with worsening surgical sepsis.

Numerous studies conducted by various authors have corroborated these findings, demonstrating that biomarker-guided therapies can reduce the incidence of mortality and illness in surgical sepsis groups [2,25,26]. A comparable investigation also corroborated our study's findings, highlighting the critical role of IL-10 in immune dysregulation and its predictive value for MODS [25]. These observations highlight the potential of IL-10 as a biomarker for stratifying sepsis severity and customizing therapeutic strategies in surgical environments.

The diverse presentation and severity of sepsis continue to pose a significant challenge in the field of critical care medicine, increasing the complexity of diagnostic and treatment procedures. The development of a universal algorithm for sepsis management is a difficult task due to the variability in incidence, symptomatology, etiology, and severity. In order to provide effective sepsis management, a multidisciplinary approach is required. A positive prognosis is contingent upon the early identification, diagnosis, and treatment of the condition. The contradictory results of multicenter trials emphasize the necessity of additional research and a collaborative approach to sepsis.

There are a few factors that may limit the ability of our research to extrapolate study findings to vast populations. The single-center design of the study poses a significant constraint in the generalizability of its findings. The cohort population of 100 patients also is not adequate enough. Multicenter studies with larger sample sizes must improve the reliability and applicability of biomarker-guided approaches in clinical practice and validate these findings across a variety of surgical sepsis cohorts. There may be a variation in healthcare settings, treatment protocols, and patient demographics, which may all have varying effects on clinical outcomes and biomarker dynamics.

## Conclusions

On analyzing our cohort population, the importance of CRP, PCT, and IL-10 in diagnosing and managing sepsis is more evident. CRP exhibited high diagnostic accuracy with 89.5% sensitivity and 99.7% specificity at a 9.35 mg/L cutoff, though its effectiveness in assessing severity was limited. In contrast, procalcitonin (PCT) levels rose significantly from admission to day 7, suggesting its utility as a marker for severity and progression. Elevated IL-10 levels were linked to severe outcomes, such as multiple organ dysfunction syndrome (MODS) and mortality, marking high-risk patients.

Sepsis is of great importance in clinical practice due to its multifaceted presentation and high mortality rates. This study seeks to emphasize the potential of biomarkers, including IL-10, CRP, and PCT, in enhancing the early diagnosis and prediction of sepsis severity in patients. The variations in these biomarkers have a strong association with clinical outcomes, underscoring their importance in guiding treatment decisions. We aim to highlight in this study the importance of having a multidisciplinary approach for effective sepsis management and improved outcomes.
